# Bevacizumab as a steroid‐sparing agent during immunotherapy for melanoma brain metastases: A case series

**DOI:** 10.1002/hsr2.115

**Published:** 2019-02-01

**Authors:** Patricia D. Banks, Arian Lasocki, Peter K. H. Lau, Shahneen Sandhu, Grant McArthur, Mark Shackleton

**Affiliations:** ^1^ Department of Cancer Medicine, Melbourne Peter MacCallum Cancer Centre Australia; ^2^ Department of Cancer Imaging Peter MacCallum Cancer Centre Melbourne Australia; ^3^ Sir Peter MacCallum Department of Oncology University of Melbourne Melbourne Australia; ^4^ Central Clinical School Monash University Melbourne Australia; ^5^ Department of Oncology Alfred Health Melbourne Australia

**Keywords:** bevacizumab, brain metastases, corticosteroids, immunotherapy, melanoma

## Abstract

**Background:**

Brain metastases are common in advanced melanoma and often necessitate corticosteroids such as dexamethasone to control symptoms and reduce peritumoral edema. Immunotherapy improves survival in metastatic melanoma, but concomitant treatment with corticosteroids may reduce efficacy. Here, we report the use of bevacizumab, a vascular endothelial growth factor (VEGF) inhibitor, as a steroid‐sparing agent in melanoma patients with brain metastases treated with immunotherapy.

**Methods:**

Medical records and imaging were retrospectively analyzed for melanoma patients with brain metastases who received bevacizumab at our institution between 2012 and 2017.

**Results:**

12 melanoma patients with brain metastases received bevacizumab (5‐7.5 mg/kg Q2‐3 W; median 4 cycles, range 1‐9). Patients were BRAF wild‐type or resistant to BRAF/MEK inhibitor therapy. All had progressive intracranial disease after prior resection, stereotactic radiosurgery and/or whole brain radiotherapy, and up to four lines of previous systemic treatment. Prior to bevacizumab, all patients had radiologically defined peritumoral edema and nine required dexamethasone for symptom control. In 10 evaluable patients, six reduced their dexamethasone dose by more than 50%, and eight displayed reduced edema 4 weeks after bevacizumab. Seven patients experienced adverse events possibly related to bevacizumab, including intracranial hemorrhage, hypertension, and gastrointestinal bleeding. Ten patients received immunotherapy after bevacizumab. Five patients survived more than 6 months, including one who remained disease‐free after 4 years and without neurological deficit despite being hemiplegic from edematous brain metastases prior to bevacizumab.

**Conclusion:**

In 12 very poor prognosis melanoma patients with brain metastases, bevacizumab was well‐tolerated, associated with improved symptoms and reduced peritumoral edema despite weaning steroids, and facilitated treatment with immunotherapy that provided durable survival in a substantial proportion of cases.

## 1 INTRODUCTION

Brain metastases are common in metastatic melanoma, with up to 20% of patients having such disease at diagnosis and up to 75% developing it over time.^1^, ^2^, ^3^ Traditionally, melanoma patients with brain metastases have had a dismal prognosis, with a median overall survival of 4.7 months^2^ and only 1.3 months for patients treated with immunosuppressive corticosteroids,^4^ which are frequently required to control tumor‐associated edema in the brain.

T‐cell checkpoint immunotherapy, such as that with anti‐CTLA‐4 (cytotoxic T‐lymphocyte‐associated protein 4) and/or anti‐PD‐1 (programmed death 1) antibodies, has dramatically improved the prognosis of patients with metastatic melanoma.^5^, ^6^, ^7^ As the mechanism of action of immunotherapy relies on robust immune responses, immunosuppression with corticosteroids may reduce the efficacy of this treatment,^8^ although this has not been conclusively demonstrated. Nonetheless, clinical trials of checkpoint inhibitors have typically excluded patients requiring more than 10 mg per day prednisolone equivalent.^7^

Concerns regarding possibly reduced efficacy of immunotherapy, as well as adverse events associated with high‐dose corticosteroids, motivate clinicians to minimize corticosteroid use in patients receiving immunotherapy. However, an increasingly common clinical dilemma arises in patients requiring steroids to control symptoms related to edema associated with brain metastases.

The mechanisms by which corticosteroids reduce vasogenic edema are not completely understood,^9^ but may be mediated by VEGF (vascular endothelial growth factor).^10^ On this basis, bevacizumab, an anti‐VEGF monoclonal antibody, has been used to improve clinical symptoms and edema associated with cerebral radiation necrosis.^11^ Interestingly, bevacizumab may be synergistic with ipilimumab against metastatic melanoma by augmenting immune cell infiltration of tumors.^12^

Bevacizumab has not previously been evaluated as a means of improving symptoms in patients with edema surrounding brain metastases. Despite this, off‐label bevacizumab has been used at our institution for this purpose in select patients with melanoma brain metastases, with the aim of minimizing steroid use and facilitating immunotherapy. Here, we present the outcomes for these patients.

## 2 METHODS

### 2.1 Patient identification and clinical data collection

The project was approved by Peter MacCallum Cancer Centre's Human Research and Ethics Committee. As this was a retrospective case series, the need for individual informed consent was waived. Pharmacy and patient records identified patients with a diagnosis of melanoma brain metastases who were prescribed bevacizumab. The medical records of these patients were retrospectively examined to obtain information about patient demographics, treatment history, clinically assessed symptoms, treatment‐associated toxicity, tumor responses, and patient survival outcomes. Adverse events were graded using the National Cancer Institute's (NCI's) Common Terminology Criteria for Adverse Events (CTCAE), version 4.03. Clinical symptoms associated with brain metastases and response after bevacizumab were gleaned from clinical notes. Data cutoff was 1 May 2017.

### 2.2 Radiology review

All cases were reviewed by a single radiologist with subspecialty expertise in neuro‐oncology. The extent of edema was measured on the axial Fluid‐Attenuated Inversion Recovery (FLAIR) sequence on magnetic resonance imaging (MRI) or, when MRI was not performed, on computed tomography (CT) in the axial plane. Temporal comparison of the extent of edema was designated as stable, increased, or decreased. This was assessed subjectively, as peritumoral edema from separate lesions was frequently confluent and could not be accurately measured. Intracranial lesions were measured on postcontrast images. The presence of intra‐ and/or extra‐tumoral hemorrhage was noted and tracked during bevacizumab therapy, using MRI where available (the susceptibility‐weighted imaging sequence), or otherwise CT. Intracranial and extracranial tumor responses to immunotherapy were assessed using RECIST 1.1 criteria.^13^

## 3 RESULTS

### 3.1 Baseline characteristics

Twelve patients diagnosed with melanoma brain metastases received bevacizumab between August 2012 and April 2017. The median age was 58 (range 32‐76). Brain metastases had been detected a median of 11 months prior to bevacizumab administration (range 2‐40 months). Patients were heavily pretreated (Table 1), having up to four (median 1) brain metastases resected and up to seven (median 0) brain metastases treated with stereotactic radiosurgery (SRS, dose range 18‐25 Gy in 1‐3 fractions). Eleven patients had previous whole brain radiotherapy (WBRT, dose range 20‐36 Gy in 5‐18 fractions) and two of these had been retreated (20 Gy in 10 fractions). All eight patients with a BRAF mutation had previously received a BRAF inhibitor, with or without a MEK inhibitor. Six patients had received ipilimumab and one of these had twice been reinduced with ipilimumab. Four patients had received PD‐1 inhibitors and four patients had received chemotherapy.

**Table 1:** Baseline characteristics

Patient	Age (y)	BRAF Status	Time Between Diagnosis Of Brain Mets And Bevacizumab (mo)	Past Treatment Of Brain Metastases			
				Surgical Resection	SRS	WBRT	Systemic Therapy
P01	40	V600E	16.2	3	2	No	BRAFi + MEKi
P02	46	V600E	3.1	0	0	Yes	BRAFi + MEKi, ipilimumab
P03	70	Wild type	3.5	1	0	Yes	Nil
P04	58	V600E	2.0	0	0	Yes	BRAFi + MEKi, temozolomide
P05	63	Wild type	2.9	0	0	Yes	Ipilimumab, temozolomide, pembrolizumab
P06	57	K601E	10.3	2	0	Yes	BRAFi, ipilimumab
P07	58	Wild type	40.3	4	6	Yes	Fotemustine, ipilimumab + 2 reinductions
P08	73	Wild type	1.6	0	0	Yes	Pembrolizumab
P09	51	V600E	22.5	1	1	Yes + retreat	BRAFi + MEKi, pembrolizumab
P10	76	V600K	11.3	0	0	Yes	BRAFi + MEKi, ipilimumab, pembrolizumab
P11	33	V600E	30.1	3	0	Yes + retreat	BRAFi + MEKi
P12	32	V600E	11.0	2	7	Yes	BRAFi + MEKi, temozolomide, ipilimumab

Abbreviations: BRAFi, BRAF inhibitor, including dabrafenib or vemurafenib; MEKi, MEK inhibitors, including trametinib or cobimetinib; SRS, stereotactic radiosurgery; WBRT, whole brain radiotherapy.

### 3.2 Bevacizumab and immunotherapy treatment

**Figure 1:**
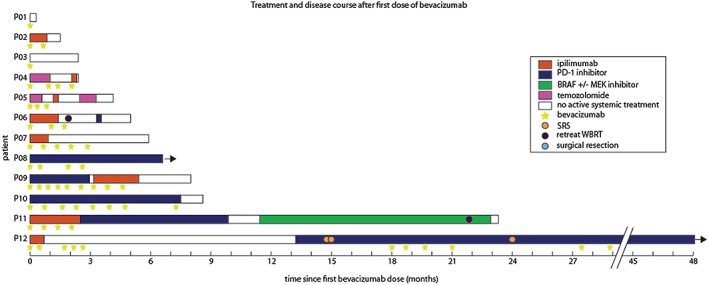
Patient timelines. Each bar represents an individual patient and starts at the first dose of bevacizumab received. Treatment received after first dose of bevacizumab is shown with colored bars indicating systemic therapy, circles indicating radiotherapy, and stars indicating doses of bevacizumab. Median survival was 5.4 months (range 9 d to more than 4 y)

Eight patients received 7.5 mg/kg intravenous bevacizumab every 3 weeks and four patients received 5 mg/kg every 2 weeks (Table 2). The median total number of bevacizumab treatments per patient was four (range 1‐9). Two patients received only one dose of bevacizumab and were not evaluable for radiological or disease outcomes; one (P01) had very rapid disease progression and died within 2 weeks, while the other (P03) declined further active treatment (Figure 1). Of the 10 evaluable patients, seven received ipilimumab and three received pembrolizumab after bevacizumab (Figure 1, Table 2). Immunotherapy and bevacizumab were given either concurrently or on separate days according to scheduling requirements. Eight patients started or continued checkpoint inhibitor immunotherapy at the time of starting bevacizumab. Two patients (P04 and P05) started temozolomide with bevacizumab while weaning corticosteroids for 5 to 9 weeks before commencing immunotherapy. Figure 1 describes systemic treatment and radiotherapy or surgery received by patients after starting bevacizumab, as well as their survival.

**Table 2:** Bevacizumab dose, steroid wean in the first 4 weeks and immunotherapy received after bevacizumab

	Bevacizumab Received^a^		Dexamethasone Dose^b^		Immunotherapy Received	
Patient	Dose	Cycles	Before	4 weeks after	Type	Cycles
P01	5 mg/kg	1	16 mg	N/A	Nil	0
P02	7.5 mg/kg Q3W	2	8 mg	0.5 mg	Ipilimumab	1
P03	7.5 mg/kg	1	0	4 mg	Nil	0
P04	5 mg/kg Q3W	4	8 mg	3.2 mg	Ipilimumab	1
P05	5 mg/kg Q2W	3	8 mg	0.5 mg	Ipilimumab	1
P06	7.5 mg/kg Q3W	3	6 mg	2 mg	Ipilimumab	2
P07	7.5 mg/kg Q3W	5	0	0	Ipilimumab	2
P08	5 mg/kg Q2W	4	2 mg	0	Pembrolizumab	10
P09	7.5 mg/kg Q2W x5 then Q3W x4	9	16 mg	4 mg	Pembrolizumab	5
P10	7.5 mg/kg Q3‐4 W	7	4 mg	0 mg	Pembrolizumab	10
P11	7.5 mg/kg Q3W	4	1 mg	1 mg	Ipilimumab	4
P12	7.5 mg/kg Q2W	5	0	0	Ipilimumab	2

a Two patients received bevacizumab again later in the course of their disease for recurrence of neurological symptoms and radiological edema with good effect without concomitant use of steroids (data not shown).

b Other corticosteroids converted to dexamethasone dose equivalent. Physiological replacement of steroids for treatment of cortisol insufficiency has been recorded as 0 mg.

### 3.3 Corticosteroid treatment

Eight of the 10 evaluable patients required dexamethasone prior to commencement of bevacizumab and six of these were able to reduce their dexamethasone dose by more than 50% in the first 4 weeks after bevacizumab (Table 2). In two others, bevacizumab was used instead of steroids to improve edema‐associated symptoms and/or to minimize potential future steroid requirements. The patient who opted against further active treatment later started steroids to improve constitutional rather than neurological symptoms. No patients treated with greater than or equal to two doses of bevacizumab required increased steroids to control brain edema.

### 3.4 Radiological findings

**Figure 2:**
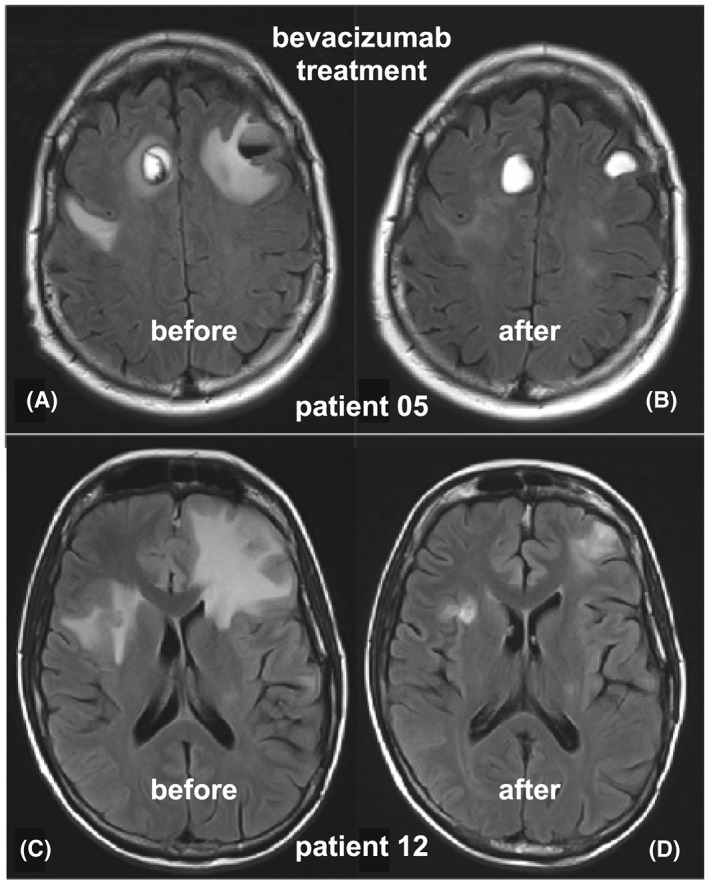
Radiological improvement in peritumoral edema after bevacizumab. Axial FLAIR images for P05 before (A) and after (B) bevacizumab show a marked decrease in the edema surrounding hemorrhagic bilateral frontal lobe metastases. The milder FLAIR hyperintensity further posteriorly was attributed to prior treatment (including WBRT). Axial FLAIR images for P12 before (C) and after (D) bevacizumab demonstrated marked improvement in edema without any use of corticosteroids

Prior to bevacizumab, all 12 patients had peritumoral edema surrounding brain metastases on imaging (Table 3), 10 with hemorrhagic changes. Patient P01 did not have follow‐up imaging after bevacizumab as he deteriorated very rapidly. Eight of the 10 evaluable patients had a decrease in peritumoral edema after bevacizumab (Figure 2, Table 3). One of these had increased hemorrhage in lesions, as well as a small asymptomatic subdural hematoma, but the surrounding edema decreased. Two patients had increased edema with progressive brain metastases. Brain metastases that were not hemorrhagic prior to bevacizumab did not become hemorrhagic after bevacizumab.

**Table 3:** Symptomatic and radiologic edema response to bevacizumab and tumor response to further antitumor therapy

Patient	Number of Brain Metastases^a^	Hemorrhage Prior To Bevacizumab^b^	Symptom Response	Edema Response	Intracranial Tumor Response^a^	Extracranial Tumor Response^c^	Overall Survival^d^
P01	5	+++	N/A^e^	N/A^e^	N/A^f^	N/A^f^	0.3
P02	≥10	+++	Worse	Worse	Stable	N/A^e^	1.5
P03	5	++	N/A^g^	Worse	N/A^f^	N/A^f^	2.4
P04	≥10	++	Stable	Mixed	N/A^e^	N/A^e^	2.4
P05	≥10	+++	Better	Better	Stable	Stable	4.1
P06	≥10	+	Stable	Better	Stable	Progression	5.0
P07	3	−	Better	Better	Stable	Progression	5.9
P08	≥10	+	Better	Better	Partial response	Complete response	>6.6
P09	≥10	++	Better	Better	Progression	Progression	8.0
P10	≥10	+	Better	Better	Progression	Stable	8.6
P11	≥10	++	Better	Better	Mixed	N/A^h^	23.3
P12	>10	++	Better	Better	Partial response	Partial response	> 48.0

Abbreviation: N/A, not applicable.

a number of brain metastases, excluding lesions possibly representing radiation necrosis or post‐operative change.

b degree of hemorrhage graded as: (−) no or minimal intralesional hemorrhage, (+) hemorrhage in a minority of lesions, (++) substantial hemorrhage in approximately half of metastatic lesions, and (+++) hemorrhage in all or most metastatic lesions.

c measured according to RECIST 1.1 where possible.

d overall survival measured in months from first dose of bevacizumab to death.

e rapid disease progression and no subsequent imaging of disease.

f patient received bevacizumab without subsequent immunotherapy.

g no neurological symptoms prior to bevacizumab therapy.

h extracranial complete response prior to bevacizumab and immunotherapy and ongoing complete response afterwards.

### 3.5 Adverse events

Adverse events potentially related to bevacizumab are described in Table 4. As above, one patient developed an asymptomatic thin subdural hematoma, which resolved without sequelae after ceasing bevacizumab. Another patient, who had received four doses of bevacizumab, had a sudden deterioration that was attributed to intracranial bleeding, although this could not be confirmed radiologically.

**Table 4:** Adverse events possibly associated with bevacizumab

Patient(s)	Adverse Event	Grade^a^	Comments
Gastrointestinal			
P05	Lower GI hemorrhage	1	Previous colitis
P07	Lower GI hemorrhage	2	Known small bowel metastases
P09	Bowel perforation	5	Known small bowel metastases and progressive disease
Nervous system			
P10	Intracranial hemorrhage	1	
P04	Intracranial hemorrhage	5	Assumed, not able to be confirmed by imaging
Vascular			
P06	Hypertension	3	Previous hypertension
Skin			
P08, P09	Dry skin	2	
P08	Wound complication	2	

a Adverse events during or after bevacizumab treatment, graded according to NCI CTCAE 4.03.

Two patients had gastrointestinal bleeding after bevacizumab in the setting of known small bowel metastases. One had had gastrointestinal bleeding before starting bevacizumab, requiring blood transfusions and palliative radiotherapy. This patient's post‐bevacizumab bleeding event self‐resolved and he received four more doses of bevacizumab without incident. The other patient developed gastrointestinal perforation and acute deterioration after eight doses of bevacizumab and progressive disease on pembrolizumab. A third patient with a history of immunotherapy‐related colitis requiring proctocolectomy developed stomal bleeding, which self‐resolved.

Exacerbation of hypertension affected one patient. Before bevacizumab, he required two anti‐hypertensive agents, but after bevacizumab (and corticosteroids), he required five medications to control blood pressure. He had multiple dose delays due to hypertension, although did not develop hypertensive complications.

### 3.6 Disease course and symptomatic responses

The median survival of the entire 12‐patient cohort from first dose of bevacizumab was 5.4 months (range 9 d to more than 4 y) (Figure 1). The median survival from diagnosis of melanoma brain metastasis was 15.9 months (range 4.5 to more than 59 mo). Of the nine patients who received more than 2 doses of bevacizumab, the median survival was more than 6.6 months (range 2.4 mo to more than 4 y). Following treatment with immunotherapy and bevacizumab, six of 10 (60%) evaluable patients obtained “intracranial benefit” (two partial responses and four stable disease by RECIST 1.1 criteria), although two of these patients had discordant responses with progressive extracranial disease. One further patient had a mixed response, with some brain metastases enlarging due to increased hemorrhage and other lesions decreasing in size. Three of 10 evaluable patients displayed intracranial disease progression despite bevacizumab and immunotherapy. Eleven of 12 patients had neurological symptoms, such as headache, nausea, or focal neurological deficits prior to commencing bevacizumab. After bevacizumab, these symptoms improved in seven evaluable patients, were stable in two patients and worsened in one (Table 3).

Two patients were retreated with bevacizumab (Figure 1). One (P10) had recurrence of neurological symptoms and edema 11 weeks after completing seven cycles of bevacizumab with pembrolizumab. He received a further dose of bevacizumab but his disease progressed rapidly and widely, and he developed bowel perforation and died.

A second patient (P12) had three separate courses of bevacizumab. Her initial bevacizumab dose was given when she became hemiplegic, dysphasic, and bedbound after her first dose of ipilimumab. The patient was keen to avoid corticosteroids and so received bevacizumab alone, with near‐complete improvement in symptoms and resolution of radiological edema. This allowed her to receive a further two cycles of ipilimumab, which was subsequently ceased because of autoimmune neutropenia. Twelve months later, she developed progressive intracranial disease and commenced anti‐PD‐1 therapy. Five months after that, she developed headaches, nausea, and increased peritumoral edema on MRI brain. Fluoro‐ethyl‐tyrosine positron emission tomography (FET‐PET) supported radiation necrosis rather than progressive tumor and she was treated with a second course of bevacizumab, with good clinical and radiological improvement. Nine months later, three brain metastases were progressing and treated with stereotactic radiosurgery (SRS). Bevacizumab was used again successfully to treat headache, dysphasia, and radiological edema. At census, the patient remained well, with ongoing anti‐PD‐1 therapy more than 4 years after her initial course of bevacizumab and had avoided corticosteroid use during this time.

## 4 DISCUSSION

Use of immunosuppressive high‐dose corticosteroids to ameliorate symptoms related to malignant progression is clinically indicated in multiple contexts, and can be life‐saving. However, this use needs to be carefully considered in the era of cancer immunotherapy, in which effective T‐cell checkpoint inhibition can lead to durable and deep antitumor responses^5^, ^6^, ^7^ against both intracranial and extracranial disease.^8^ The CheckMate 204 and Australian ABC studies demonstrated intracranial response rates of 44% to 55% with combination of ipilimumab/nivolumab immunotherapy in patients with asymptomatic melanoma brain metastases,^14^, ^15^ although response rates in symptomatic disease appear to be lower.^15^ As a result of immunotherapy, cancer patients with previously very poor prognosis disease, such as brain metastases from melanoma, may now live many years.^6^, ^16^ This highlights the importance of minimizing immunosuppression and maximizing opportunities for effective immunotherapy in every patient.

We have found that use of bevacizumab can be highly effective in the control of peritumoral edema from brain metastases in melanoma patients. It permits weaning of steroids while improving edema and symptoms, facilitating treatment with immunotherapy that can provide durable control of disease in otherwise very poor prognosis contexts.^4^ Bevacizumab has previously and successfully been used to wean corticosteroids in patients with symptomatic cerebral radiation necrosis^11^ and has been shown to improve radiological edema and clinical symptoms associated with radiation necrosis in melanoma brain metastases.^17^ Our findings show that its use can be safely and efficaciously extended to patients with progressing, symptomatic, and edematous brain metastases, particularly when immunotherapy is planned.

Most patients in our series had heavily pretreated brain metastases, including surgical resections, radiotherapy, and previous systemic treatment. As such, they constituted a subset of patients with particularly poor prognosis and limited treatment options. Bevacizumab was used in these patients with the aim of reducing corticosteroid requirements and, thereby, maximizing the likelihood of tumor response to subsequent immunotherapy.

Furthermore, there may be synergistic effects between bevacizumab and checkpoint inhibitors. The addition of bevacizumab to ipilimumab was associated with increased immune cell trafficking, increased circulating memory T‐cells, and changes in tumor vasculature,^12^ potentially enhancing antitumor effects of immunotherapy. Bevacizumab in combination with temsirolimus was associated with reduced circulating FoxP3+ regulatory T‐cells compared with pretreatment samples.^18^ The addition of bevacizumab to carboplatin and paclitaxel chemotherapy appeared to improve survival and response rates, but this was not statistically significant.^19^ These studies were performed in patients with advanced melanoma but excluded brain metastases. There are a number of current clinical trials investigating bevacizumab in combination with anti‐PD‐1/PD‐L1 antibodies in advanced melanoma, including one specifically for untreated brain metastases and including symptomatic patients requiring corticosteroids (NCT03175432). Our case series highlights the importance of these studies.

In our series, bevacizumab was used as a steroid‐sparing agent and the majority of patients had improved neurological symptoms and a reduction in steroid dose during commencement of immunotherapy. In other patients, bevacizumab prevented the need to introduce steroids at all, while still maintaining patient well‐being during immunotherapy. Apart from one very early death due to rapid disease progression, the 10 patients who wished for ongoing treatment received immunotherapy. Without bevacizumab, these patients may have required continued high‐dose steroids, incurring potential side effects and possibly reducing efficacy of immunotherapy. Prospective trials evaluating the addition of bevacizumab to corticosteroids alone should ascertain whether bevacizumab facilitates steroid weaning.

Other studies investigating antitumor activity of bevacizumab demonstrated its safety in patients with brain metastases from solid tumors. The BRAIN study investigated 91 patients with nonsquamous nonsmall cell lung cancer and asymptomatic brain metastases.^20^ This study focused on patients with little or no edema related to their metastases, therefore limiting detection of a potential effect of bevacizumab in decreasing edema.^20^ The REBECA study used bevacizumab in combination with whole brain radiotherapy for brain metastases from breast, lung, ovarian, or unknown primary tumors.^21^ All patients were started on prophylactic steroids prior to treatment.^21^ None of these studies assessed the efficacy of bevacizumab in reducing peritumoral edema and resultant symptoms, or in enabling steroid wean. However, they did conclude that bevacizumab could be safely used in patients with brain metastases.

Of particular concern in patients with brain metastases is the risk of tumor‐associated bleeding, which may be promoted by bevacizumab due to poorly understood mechanisms that may include reduced renewal of endothelial cells and consequent compromise of blood vessel integrity.^22^ In the BRAIN study, one patient (1%) had a grade 1 intracranial hemorrhage that resolved without sequelae.^20^ In the REBECA trial, two of 19 patients (11%) had intralesional hemorrhage but no parenchymal brain hemorrhage.^21^ In glioblastoma multiforme, bevacizumab was associated with a higher rate of intracranial hemorrhage than steroids alone (3.3% vs 2%).^23^

Hemorrhage is of particular concern in melanoma brain metastases because of their high propensity for spontaneous hemorrhage.^24^, ^25^, ^26^ Consistent with this, 10 of 12 patients in our series had brain metastases with imaging evidence of hemorrhage prior to treatment. Despite this, and given the inherent tendency of melanoma metastases to bleed, no patients had radiologically confirmed worsening of intratumoral hemorrhage that could be specifically attributed to bevacizumab. However, it is difficult to exclude an effect of bevacizumab on hemorrhage risk given the above, so the risk of catastrophic hemorrhage should be discussed with patients ahead of treatment.

Gastrointestinal perforation may also complicate therapy with bevacizumab.^22^ In our series, two patients with known small bowel metastases experienced gastrointestinal bleeding during bevacizumab treatment, including one receiving concurrent pembrolizumab complicated by a fatal bowel perforation. Although gastrointestinal metastases are common in patients with melanoma, spontaneous bleeding and perforation are not.^27^ It is unknown whether immunotherapy‐associated colitis might be more inclined to be complicated by perforation when treated concurrently with bevacizumab. Again, although gastrointestinal complications may occur spontaneously, the small but life‐threatening risk of perforation should be discussed ahead of bevacizumab treatment in melanoma patients, particularly those with known bowel metastases or a history of enterocolitis.

The main limitations of our study are its small, retrospective, single‐center nature, and our lack of a control comparator group. This is related to our off‐label use of bevacizumab in this context, which necessitated self‐funding by patients and limited development of the patient cohort. This also explains the heterogeneity in patient management in our cohort, including dosing schedules, as some patients elected to have lower dose treatment with 5 mg/kg, which regardless proved adequate.

Nevertheless, our description of the use of bevacizumab as a steroid‐sparing agent in 12 patients with edematous and heavily pretreated melanoma brain metastases provides a compelling rationale for prospective testing of this approach in clinical trials. In this very poor prognosis context, we found that bevacizumab was effective in improving neurological symptoms and reducing peritumoral edema, thus enabling patients to receive immunotherapy while minimizing immunosuppression from corticosteroids. Although this approach offers such patients the chance of long‐term disease control by facilitating maximally effective immunotherapy, patients should be informed of potential safety concerns and the current lack of defined benefit from prospective trials.

## FUNDING

This work was not supported by specific funding.

## CONFLICTS OF INTEREST

The authors declare no conflicts of interest.

## AUTHOR CONTRIBUTIONS

Conceptualization: Mark Shackleton, Grant McArthur, Shahneen Sandhu

Data Curation: Patricia Banks, Arian Lasocki, Peter Lau

Formal Analysis: Patricia Banks, Arian Lasocki, Peter Lau, Mark Shackleton

Investigation: Patricia Banks, Arian Lasocki, Peter Lau

Supervision: Mark Shackleton, Grant McArthur, Shahneen Sandhu

Visualization: Patricia Banks, Arian Lasocki, Shahneen Sandhu, Mark Shackleton

Writing – Original Draft: Patricia Banks, Arian Lasocki, Mark Shackleton

Writing – Revision and Editing: Patricia Banks, Arian Lasocki, Grant McArthur, Shahneen Sandhu, Mark Shackleton.
